# Superinfection between Influenza and RSV Alternating Patterns in San Luis Potosí State, México

**DOI:** 10.1371/journal.pone.0115674

**Published:** 2015-03-24

**Authors:** Jorge Xicoténcatl Velasco-Hernández, Mayra Núñez-López, Andreu Comas-García, Daniel Ernesto Noyola Cherpitel, Marcos Capistrán Ocampo

**Affiliations:** 1 Instituto de Matemáticas, Universidad Nacional Autónoma de México, Boulevard Juriquilla No. 3001, Juriquilla, 76230, México; 2 Departamento de Matemáticas Aplicadas y Sistemas, DMAS, Universidad Autónoma Metropolitana, Cuajimalpa, Av. Vasco de Quiroga 4871, Col. Santa Fe Cuajimalpa, Cuajimalpa de Morelos, 05300, México, D.F., México; 3 Facultad de Medicina, Universidad Nacional Autónoma de México, México, Av. Universidad 3000, CP 04510, Mexico City, Mexico; 4 Departamento de Microbiología, Facultad de Medicina, Universidad Autónoma de San Luis Potosí, Av. Venustiano Carranza 2405, CP 78210, San Luis Potosí, México; 5 Centro de Investigación en Matemáticas A.C., Jalisco S/N, Col. Valenciana, 36240, Guanajuato, Gto., México; Alberta Provincial Laboratory for Public Health/ University of Alberta, CANADA

## Abstract

The objective of this paper is to explain through the ecological hypothesis superinfection and competitive interaction between two viral populations and niche (host) availability, the alternating patterns of Respiratory Syncytial Virus (RSV) and influenza observed in a regional hospital in San Luis Potosí State, México using a mathematical model as a methodological tool. The data analyzed consists of community-based and hospital-based Acute Respiratory Infections (ARI) consultations provided by health-care institutions reported to the State Health Service Epidemiology Department from 2003 through 2009.

## Introduction

Influenza and respiratory syncytial virus (RSV) are leading etiologic agents of acute respiratory infections (ARI). These viruses are associated to significant morbidity, mortality and school/work absenteeism during winter season [1–3]. Every year, influenza infects approximately 20% of children and 5% of adults [4], while RSV infects almost all the children during the first three years of life; re-infections by these viruses are very common during early childhood [5]. In addition, RSV is currently recognized as an important pathogen for all age groups, including the elderly [6–8]. In temperate climates influenza and RSV occur as annual epidemics during the winter season. Each year the magnitude and timing of ARI epidemics varies. In several regions of the world a biennial RSV circulation pattern has been reported, with alternating short and long inter-epidemic periods [9–11]. We have previously reported that RSV and influenza exhibit an alternating pattern in San Luis Potosí (SLP) [12]. Variations in these epidemiological patterns could be the result of the presence of more than one circulating respiratory virus or more than one circulating influenza strain at the same time [3]; in addition, climatic variability is a driving force and host availability is a limiting resource, whose role must be explored as they affect the observed epidemic patterns for both influenza and RSV. Previous work has shown that viral interference may affect the spread of influenza. In Sweden, a rhinovirus epidemic that occurred after the end of the summer holidays may have interfered with the spread of pandemic influenza [13]. Also, epidemiological studies have suggested that interference between RSV and influenza may occur; several reports have described the interruption of RSV circulation by influenza epidemics [14, 15]. Nishimura et al. found that interference between RSV and influenza did not affect the clinical severity of the RSV epidemic [15]. In vitro studies have shown that previous influenza infection results in a reduction in RSV replication [16]. In a mouse model, influenza infection prior to RSV infection resulted in a reduction in recruitment of inflammatory cells and cytokine secretion, as well as protection against clinical symptoms associated to the infection [17]. The objective of this paper is to explain through an ecological hypothesis of competitive interaction between both viral populations and niche (host) availability, the alternating patterns of RSV and influenza observed in the data using a mathematical model as a methodological tool.

## Materials and Methods

### Data

**Fig 1:**
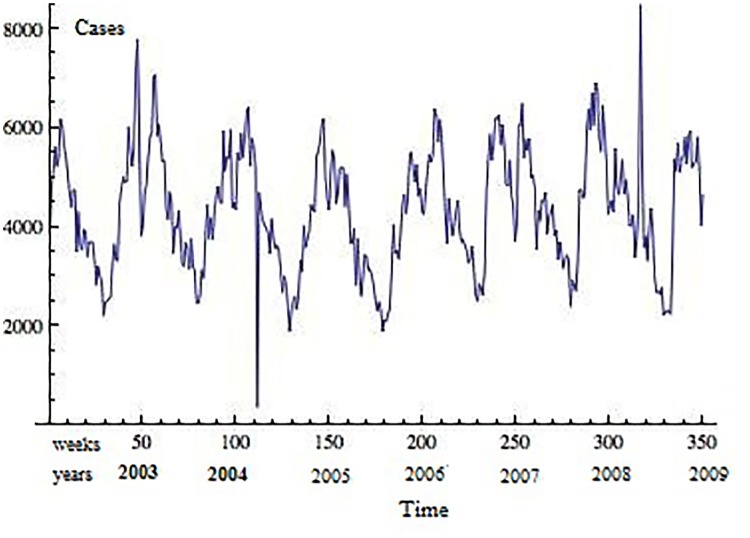
Weekly number of acute respiratory infections reported to the State Health Services Epidemiology Department, San Luis Potosí, México (2003–2009).

The data analyzed in this work consists of community-based and hospital-based ARI consultations provided by health-care institutions in the State of San Luis Potosí and reported to the State Health Service Epidemiology Department from 2003 through 2009. The weekly number of consultations provided by all institutions was registered according to the International Classification of Disease, 10th review. For this study we included respiratory infections classified with ICD-10 codes: J00-J06, J20, J21, except J02.0 and J03.0 [12]. The time series for ARI cases is presented in Fig. 1. The State of San Luis Potosí is located in Central Mexico and, according to the 2010 census, has a population of 2,585,518. San Luis Potosí City is the largest city and capital of the state, with a population in its metropolitan area of 1,040,443 [18].

**Fig 2:**
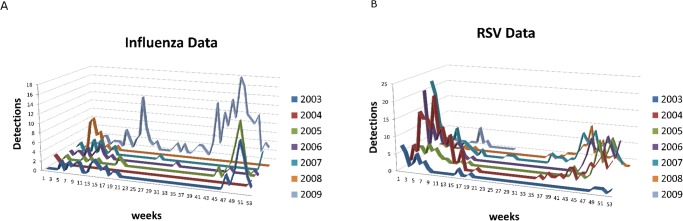
Data 2003–2009 from the records maintained by the Virology Laboratory of the Facultad de Medicina, UASLP, San Luis Potosí, Mexico. (**A**) Weekly influenza. (**B**) Weekly RSV. Each individual year is plotted in a separate graph.

RSV and influenza data was obtained from the records maintained by the Virology Laboratory (Facultad de Medicina, UASLP, San Luis Potosí, Mexico) [12, 19–20]. The time series for influenza and RSV infections are shown in Fig. 2. Most of the samples submitted to our laboratory during the study period were obtained from children under 5 years of age evaluated at the Emergency Department or admitted to Hospital Central “Dr. Ignacio Morones Prieto”.

The Hospital Central “Dr. Ignacio Morones Prieto” operates as a pediatric sentinel detection center of influenza and RSV. Due to the patients age there might be a potential sampling bias (RSV detection will be higher than influenza detections), but despite that, detection records are very useful to determine the period of circulation of both viruses and can be extrapolated to all the population. First, since the Hospital Central “Dr. Ignacio Morones Prieto” is the most important hospital integrating reports in the State of San Luis Potosí, the respiratory viruses detected due to the pediatric surveillance viral respiratory program are representative of what happens (in terms of viral circulation) throughout the all state. Second, although the majority of the viruses are detected in children under the age of 5 (that includes hospitalized and ambulatory patients), the circulation patterns of the viruses correlates in time with the seasonal pattern of acute respiratory infections outbreaks that occurs throughout the state and for all groups of age (is noteworthy that the majority of the ARI registers happen in ambulatory patients). Previously we have observed that when RSV predominates the number influenza detections is low and vice versa; despite our sampling bias, when influenza circulates in the population it is detected among all age groups [21]. Moreover, Munywoki and Nokes [22] reported that the beginning of an RSV outbreak (that affect other group ages) start in children under 5 years of age. Those results are indicative that the presence of RSV in infants could be and is in fact an indicator of RSV circulation in other age groups. Regarding the alternating influenza/RSV outbreaks patterns in older populations, there is a notable lack of information in the literature. A few studies from Douglas M. Fleming in UK have shown the same alternating pattern of influenza/RSV or pneumonia/bronchitis (the first infection was attributable to Influenza and the second to RSV) in elderly population [7, 23–25].

This study included retrospective analyses of information available from databases(weekly number of consultations associated to respiratory infections and weekly number of viral detections). All information used in this analysis was anonymized. Data used for this analysis included only weekly number of cases and, therefore, review by an ethics committee was not requested. Weekly data for acute respiratory infection- associated consultations is recorded as part of routine surveillance activities carried out by the State Public Health Department. Virological information was derived from different projects carried out to analyze the epidemiology of viral respiratory infections and as part of the hospital’s infection control program; the research projects were approved by the Research and Ethics Committee at Hospital Central “Dr. Ignacio Morones Prieto” and informed consent was obtained from children’s parents.

### Scalograms for total ARI, influenza and RSV

There are several factors that influence the ARI dynamics like environmental and climate factors, host-pathogen interactions and immunological factors. Influenza and RSV data show nonstationarity, oscillations and seasonality, features that imply that wavelet analysis may be efficient to identify patterns driven by the annual weather cycle. Unlike Fourier analysis wavelet analysis is able to analyse signals with changing spectra allowing the estimation of the spectral characteristics as a function of time [26].

Wavelets constitute a family of functions that depends on two parameters, one for time position and the other for the scale and related to the frequency. In the literature there are many studies of “natural signals” where the so-called Morlet wavelet has found wide application in diverse fields of sciences among them epidemiological time series [26–28].

The continuous wavelet transform (CWT) is shown in scalograms where the absolute value of the wavelet transform is plotted so scalograms reflect only the “power” of the signal and not the phase characteristics of the oscillatory behavior. Wavelets constitute a family of functions derived from a single function Ψ_*a*, *τ*_(*t*), that can be expressed as the function of two parameters, one for the time position *τ*, and the other for the scale of the wavelets *a*, related to the frequency. More explicitly, wavelets are defined as

$$\Psi_{a,\tau }\left(t\right)=\frac{1}{\sqrt{a}}\Psi \left(\frac{t-\tau }{a}\right).$$

We applied wavelet spectral techniques, whose graphical representations is shown here through scalograms. For wavelet analysis we use the Morlet wavelet with *α* = 5% significance level (95% confidence level).

**Fig 3:**
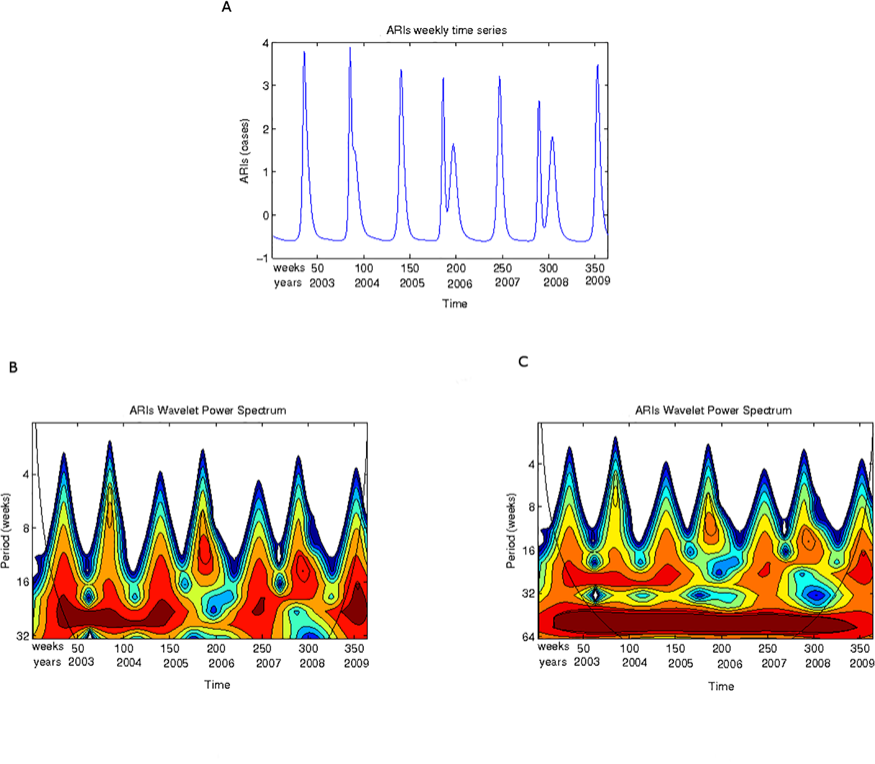
Acute respiratory infections. **(A)** The acute respiratory infections time series (2003–2009) used for the wavelet analysis. **(B)** Scalogram (absolute values) for total ARIs for the years 2003–2009 for periods between 4 and 32 weeks. **(C)** Scalogram (absolute values) for total ARIs for the years 2003–2009 for periods between 8 and 64 weeks. Horizontal axis, weeks numbered consecutively starting 2003; vertical axis, periods in weeks. The colours code for power values from white for low CTW power to dark red for high CWT power.

**Fig 4:**
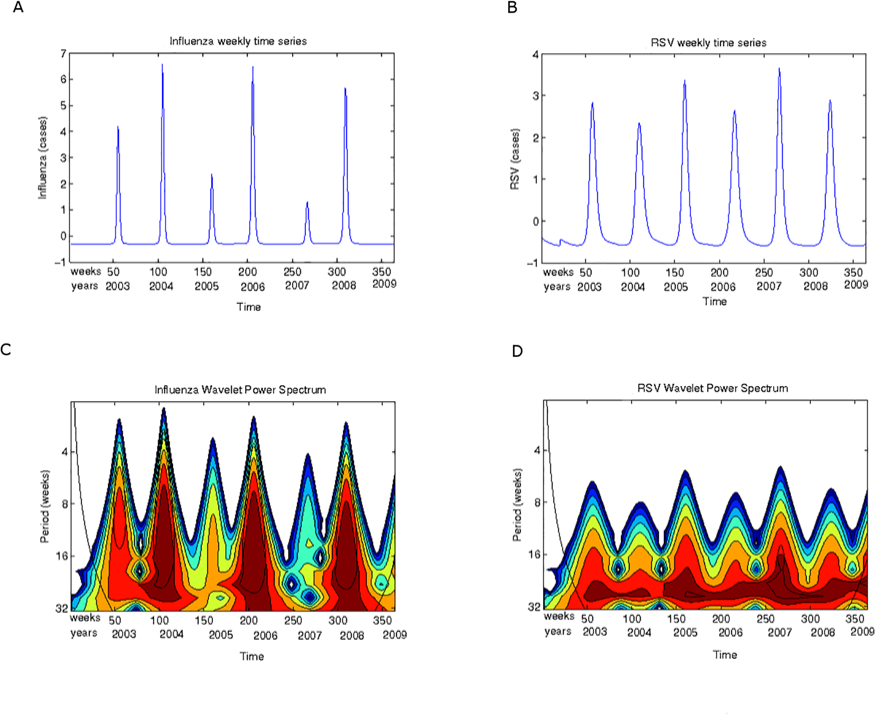
RSV and Influenza epidemics. **(A)** The Influenza time series (2003–2009) used for the wavelet analysis. **(B)** The RSV time series (2003–2009) used for the wavelet analysis. **(C)** Scalogram (absolute values) for total influenza cases for periods between 4 and 32 weeks. **(D)** Scalogram (absolute values) for total RSV cases for periods between 4 and 32 weeks. Horizontal axis, weeks numbered consecutively starting 2003; vertical axis, periods in weeks.

**Fig 5:**
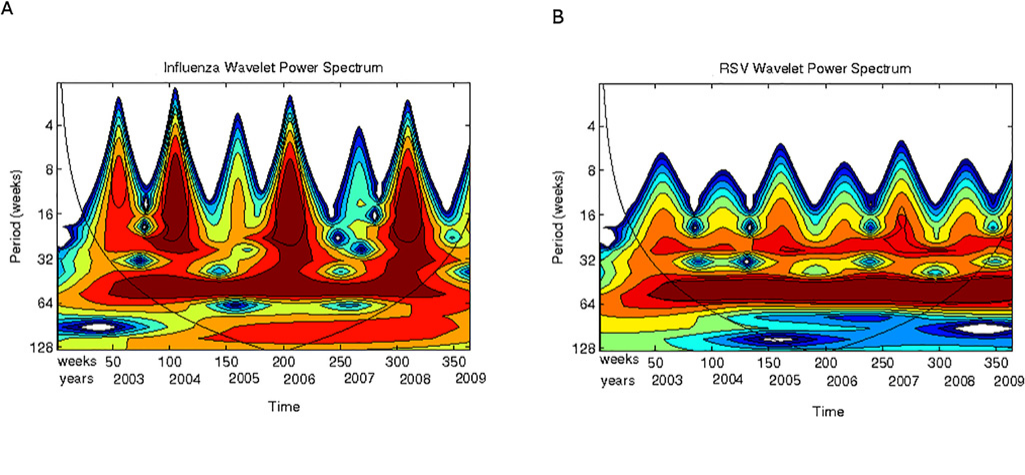
Scalograms (absolute values) for periods between 8 and 64 weeks (2003–2009). **(A)** Total influenza cases. **(B)** Total RSV cases. Horizontal axis, weeks numbered consecutively starting 2003; vertical axis, periods in weeks.

In Figs. 3 to 5 the scalograms for the years 2003 to December 2009 are shown for ARIs, influenza and RSV respectively, in two different scale ranges. One range corresponds to lengths up to 32 weeks; the second range corresponds to lengths up to 64 weeks.

In Fig. 3C the ARIs annual 52 week period is easily observed which dominates all other frequencies. Looking at lower periods (Fig. 3B), another weaker periodic cycle between 16 and 32 weeks (average of 6 months) can be seen across all weeks. Apart from this two periodic epidemic events (yearly and roughly 6-month cycles), there are activity periods of lengths smaller that 8 weeks occurring not very regularly along the years. There are very noticeable epidemic outbreaks at the end of 2003, beginning of 2005, beginning of 2007 and a very strong signal in the first quarter of 2009. One should also notice the high power signal that appears from mid 2007 to mid 2008 in the periods between 16 and 32 weeks.

The data base allows independent analysis of RSV and influenza epidemics. Fig. 4 shows this comparison for the periods between 4 and 32 weeks. It is evident that influenza epidemic events are more sparse that RSV epidemics probably due to the lower incidence of influenza in children. From 2003–2009 only four influenza epidemic events show in the scalogram (end of 2003, end of 2005, end 2007 and most of 2009, see Fig. 4C); however, RSV epidemics events occur roughly every 6 months with some years showing two epidemics (2003, 2005, 2007, 2009, see Fig. 4D). Moreover, the influenza epidemic events are relatively sharp and occur every two years (vertical fingers in Fig. 4C) as compared to the more variable event strength in RSV epidemics (multiple fuzzy, “broken” fingers in Fig. 4D) that occur every year.

In Fig. 5 scalograms for higher scales are presented (8 to 64 weeks). Here an interesting observation can be made: RSV signal power occurs between high power peaks in influenza. Both signals show a strong annual periodic signal across all weeks being stronger for RSV than for influenza. A lower power cycle in the scale between 16 and 32 weeks also occurs (see the continuous orange band across all weeks). This implies that the high power signal shown in dark red in Fig. 3C) is mainly driven by RSV. Thus RSV could be thought of as an endemic, competitively dominant virus in the San Luis population; and influenza a competitively inferior one that survives at low frequencies with significant epidemic events occurring roughly every 2 years.

Also note (Fig. 4) that at weeks 150 and 250 there are two influenza epidemic outbreaks and that between them there is almost no influenza activity; however, RSV activity does occur between weeks 150 and 250. Thus at periods below 32 weeks, RSV and influenza epidemic events alternate.

Looking at the total ARIs scalogram one can appreciate that the dynamics described in the previous paragraph is only weakly identifiable when looking at the scale of a year (Fig. 3C) but that focusing on smaller periods this interesting replacement/interference dynamics is neatly observed.

**Fig 6:**
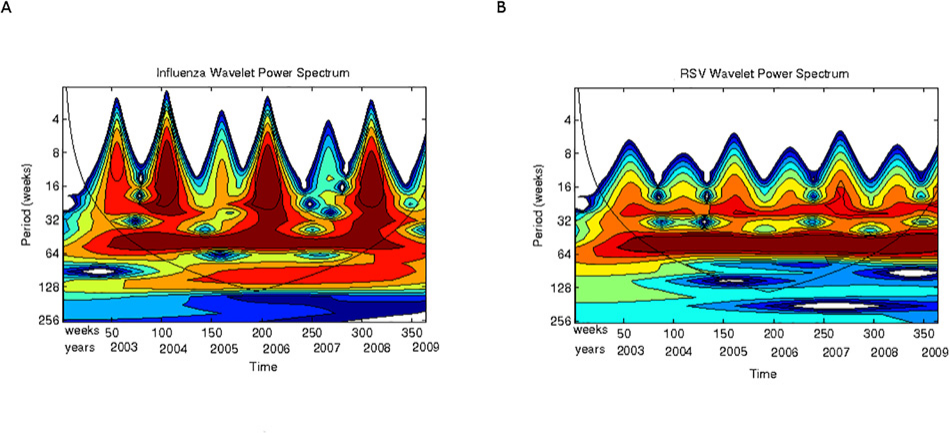
Scalograms (absolute values) for the periods between 8 and 128 weeks (2003–2009). **(A)** Total influenza cases. **(B)** Total RSV cases. Horizontal axis, weeks numbered consecutively starting 2003; vertical axis, periods in weeks.

Finally we show a very interesting phenomenon. We performed the scalogram analysis looking for periodic cycles of longer scales. Fig. 6 shows the results. One can see that influenza has a cyclic periodic behavior across weeks of about 128 weeks (32 months or roughly 3 years).

### Mathematical model

Superinfection is a long standing hypothesis that explains the coexistence of species in a given common environment [29–30]. In the absence of superinfection, a measure of the competitive ability of the most virulent strain, competitive exclusion of the weaker strain is the generic outcome [31]. Levin and Pimentel [32] were the first to show that the inclusion of superinfection makes coexistence possible. Superinfection establishes a competitive hierarchy driven by the abilities of each species to use common resources. Moreover, it is known that heterogeneities in hosts characteristics, in this case driven by climate variability and temporal immunity, are important factors for the coexistence of species [33]. Below we present a simple model for the interaction of RSV and influenza viruses based upon superinfection as a mechanisms to explain the patterns described in the previous section.

The model is a SEIRS epidemic model coupled through superinfection. RSV has reported a slightly higher *R*_0_ or reproductive number than influenza (*R*_*i*_ = 1.6 and *R*_*r*_ = 1.7 where *i* stands for influenza and *r* for RSV, [34] but this difference is rather small and the uncertainties in the estimation of both reproductive numbers may blur the difference. Therefore, we have approximated the estimates of the reproduction numbers using the data for SLP as described in the following section. The reason for the choice of a SEIRS model is that we are following seasonal influenza (and RSV) and therefore we need to account for some immunity left by any given epidemic episode in the population.

The model stands as follows. Let the subindex *k* = 1, 2 represent the two different infections in a host human population in demographic equilibrium whose size has been rescaled to *N* = 1. The larger subindex indicates superior competitive ability. Then we have

$$\begin{array}{ccc} \frac{d}{dt}S & = & \mu N-S\left(b_{1}I_{1}+b_{2}I_{2}\right)/N-\mu S+\theta_{1}R_{1}+\theta_{2}R_{2}\\ \frac{d}{dt}E_{1} & = & Sb_{1}I_{1}/N-\sigma b_{2}E_{1}I_{2}/N-\left(\mu +\gamma_{1}\right)E_{1},\\ \frac{d}{dt}I_{1} & = & \gamma_{1}E_{1}-\sigma b_{2}I_{1}I_{2}/N-\left(\mu +\eta_{1}\right)I_{1},\\ \frac{d}{dt}R_{1} & = & \eta_{1}I_{1}-\sigma b_{2}R_{1}I_{2}/N-\left(\mu +\theta_{1}\right)R_{1},\\ \frac{d}{dt}E_{2} & = & Sb_{2}I_{2}/N+\sigma b_{2}\left(E_{1}+I_{1}+R_{1}\right)I_{2}/N-\left(\mu +\gamma_{2}\right)E_{2},\\ \frac{d}{dt}I_{2} & = & \gamma_{2}E_{2}-\left(\mu +\eta_{2}\right)I_{2},\\ \frac{d}{dt}R_{2} & = & \eta_{2}I_{2}-\left(\mu +\theta_{2}\right)R_{2}, \end{array}$$ (1)

where *b*_*k*_(*t*) = *f*(*t*)*β*_*k*_ is the time-dependent infection rate for each of the infections, respectively, described in S1 File; *f*(*t*) is a function associated to climate variability and *β*_*k*_ defined as the product of the infection probability by the per capita number of contacts per unit time is the constant contact rate for virus *k*. Susceptible, exposed, infectious and immune hosts for each of the infections are represented, respectively, by *S*, *E*_*k*_, *I*_*k*_ and *R*_*k*_ with *μ* the natural mortality rate of the population. In the equations, *σ* measures a reduction in susceptibility after primary infection since an exposed, infectious or immune individual takes preventive actions against a future infection. The parameters *γ*_*k*_ and *η*_*k*_ are, respectively, the incubation and infection recovery rates for each disease; *θ*_*k*_ is the waning of immunity rate, RSV immunity takes around 3–5 years to become protective which differs from influenza which is much faster.

As it can be seen we have established that the secondary infection can take over hosts from any of the compartments at a rate *σβ*_2_; so stages *E*_1_, *I*_1_ and *R*_1_ of the primary infection become *E*_2_ as soon as they are infected. The existence of equilibrium points as a function of the reproduction numbers is shown in Figure A in S2 File.

### Parameter estimation

The objective of this section is compute two main parameters for our model: a) the reproduction number and b) the forcing function associated to climate variability. Once the estimate of each reproduction number is obtained, we get the average value for *β*_*k*_ for each virus and this value is then plugged into the model to generate our simulations. As it is well known, the reproduction number, denoted by *R*_0_, is the expected number of secondary cases produced in a susceptible population by a typical infective individual during the time in which s/he is infectious. If *R*_0_ < 1, then on average an infected individual produces less than one new infected individual over the course of its infectious period and the infection cannot grow. Conversely if *R*_0_ > 1 then each infected individual produces, on average more than one new infection and the disease can invade the population. From its definition *R*_0_ is determined from early stages of the epidemic. The information that is most commonly used to estimate *R*_0_ are reported cases and sero-prevalence data *i.e.* data on hosts who have antibodies against the pathogen. On the other hand, the replacement number [35] is defined as the expected number of secondary infections that one infected person would produce through the entire duration of the infectious period where the population need not be fully susceptible,

$$R_{e}\left(t\right)=R_{0}s\left(t\right)$$

where *s*(*t*) = *S*(*t*)/*N*. When *R*_*e*_ < 1 for *R*_0_ > 1, either through an increase in the infectious population or the recovered population through recovery from infection or vaccination, the disease can no longer sustain itself and will die out or epidemics will not occur.

In our case we have confirmed cases for both RSV and influenza from a hospital-based consultation. To calculate the basic reproduction number we rearranged the model so that the relevant quantity *R*_0_ could be obtained from linear regression.

To estimate the reproduction number by using of the classical Kermack-McKendrik SIR model we first estimated the “growth rate” by fitting an exponential function to the early ascending phase of daily infections where the epidemic curve is based on new cases onset [36],

$$I\approx I_{0}e^{\left(\left(R_{0}-1\right)\left(\eta +\mu \right)t\right)}$$

where *I*_0_ is the initial number of infectives, 1/*η* is the infectious period, and 1/*μ* is the host lifespan.

Therefore we can estimate *R*_0_ from the initial increase of infected cases. The purpose of this section is to determine the value of *R*_0_ for both respiratory diseases: Influenza and RSV. For this we need the parameters *γ*_*k*_ and *η*_*k*_ the incubation and infection recovery rates, respectively, for each disease (see Table 1).

**Table 1:** The incubation and infection recovery rates for influenza and RSV [36].

**Parameter**	**Value (*i* = influenza; *r* = RSV)**
*γ*_*i*_	0.5*d* ^−1^
*γ*_*r*_	0.2*d* ^−1^
*η*_*i*_	0.25*d* ^−1^
*η*_*r*_	0.2*d* ^−1^
*μ*	0.000039*d* ^−1^

This method for the estimation of *R*_0_ has several drawbacks, one of them being that the number of infected hosts is very low at the beginning of an epidemic. However a way around this obstacle is to use data from multiple outbreaks, so that we obtain an average basic reproductive number.

To complement these calculations we use the exponential growth rate method (EG) of the Package *R*0 [37], a language R toolbox to estimate reproduction numbers for epidemic outbreaks. During the early phase of an outbreak the exponential growth rate is defined by the per capita change in number of new cases per unit of time, as incidence data are integer valued, the EG method uses a Poisson regression to estimate this parameter rather than linear regression of the logged incidence [38].

In what follows we show the *R*_0_ value for each of the years for which data is available. For influenza and RSV we analyze data from 2003 to 2009.

### Estimating *R*_0_ for influenza and RSV

In Fig. 2 we show the influenza and RSV cases by week from 2003–2009. Note that epidemics are generally at the end or beginning of a year. For the case of influenza we calculate *R*_0_ for the individual outbreaks occurring in the winter seasons.

In Tables 2–3 we list the estimated values of *R*_0_ for each of these outbreaks.

**Table 2:** First outbreak of influenza. The *t*-test statistically significant *p* < 0.05 is marked with **.

Year	*R*_0_ (SIR)	*β* (SIR)	*R*_0_ (EG)	Confidence interval	*β*
2003	4.24**	1.06	1.11	[0.96, 1.30]	0.28
2004	—	—	—	—	—
2005	—	—	—	—	—
2006	3.67	0.92	1.09	[0.84, 1.44]	0.27
2007	2.46	0.62	1.05	[0.76, 1.43]	0.26
2008	2.21**	0.55	0.60	[0.23, 1.04]	0.15
2009	7**	1.75	1.58	[1.10, 2.31]	0.40

**Table 3:** Second outbreak of influenza. The *t*-test statistically significant *p* < 0.05 is marked with **.

Year	*R*_0_ (SIR)	*β* (SIR)	*R*_0_ (EG)	Confidence interval	*β*
2003	2.67**	0.67	1.57	[0.70, 2.65]	0.39
2004	—	—	—	—	—
2005	2.97**	0.74	1.75	[1.10, 2.55]	0.44
2006	—	—	—	—	—
2007	—	—	—	—	—
2008	—	—	—	—	—
2009	6.09**	1.52	3.19	[2.45, 4.10]	0.80

For the outbreaks during 2004, the first outbreak of 2005 and the second outbreak in the period 2006–2008 it was not possible calculate the *R*_0_ because of the scarcity of data.

For RSV Tables 4 and 5 show the estimates of the reproduction number. As influenza case, there is not enough information for some years.

**Table 4:** First outbreak of RSV. The *t*-test statistically significant *p* < 0.05 is marked with **.

Year	*R*_0_ (SIR)	*β* (SIR)	*R*_0_ (EG)	Range	*β*
2003	—	—	—	—	—
2004	2.28**	0.46	1.59	[1.09, 2.14]	0.32
2005	—	—	—	—	—
2006	—	—	—	—	—
2007	—	—	—	—	—
2008	—	—	—	—	—
2009	4.75	2.38	1.33	[0.97, 1.85]	0.27

**Table 5:** Second outbreak of RSV. The *t*-test statistically significant *p* < 0.05 is marked with **.

Year	*R*_0_ (SIR)	*β* (SIR)	*R*_0_ (EG)	Range	*β*
2003	2.26**	0.47	0.56	[0.18, 1.21]	0.11
2004	8.9**	1.78	1.83	[1.44, 2.32]	0.37
2005	4.07**	0.82	2.75	[1.89, 3.93]	0.55
2006	2.45**	0.49	1.97	[1.13, 3.27]	0.39
2007	5.78**	1.16	1.78	[1.44, 2.21]	0.36
2008	6.32**	1.26	1.35	[1.09, 1.65]	0.27
2009	—	—	—	—	—

Tables A-D in S1 File show the estimated values of R0, the estimation error and p-value for Influenza and RSV using linear regression. Acute respiratory infections are endemic in San Luis Potosí and thus the reproduction number must be near one (see Table E in S1 File).

**Fig 7:**
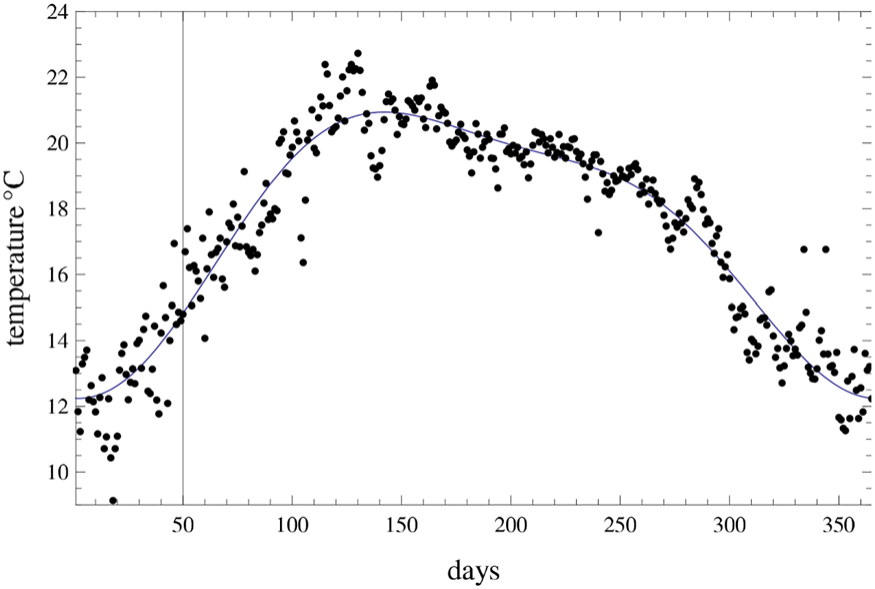
Average daily temperature data in SLP. Daily record from 2003 to 2009.

To end this section, *f*(*t*) is the forcing associated to climate variability and was estimated from the daily average temperature data for San Luis Potosí for the time period corresponding to the epidemic data (Fig. 7) resulting in the function

$$f\left(t\right)=17.33+0.480\sin\left[\frac{2\pi }{365}t\right]-0.40\sin\left[\frac{2\pi }{365}2t\right]-4.03\cos\left[\frac{2\pi }{365}t\right]-1.05\cos\left[\frac{2\pi }{365}2t\right].$$

In the mathematical model *b*_*k*_(*t*) = *f*(*t*)*β*_*k*_ is the time-dependent infection rate for each of the infections so that in the model simulation the forcing function with annual periodicity multiplies the contact rate *β*_*k*_.

## Results

As [29–30] have pointed out, virulence and superinfection play an essential role on the evolution of pathogens. Host demography has a very significant role on the long-term interaction of hosts and pathogens and it is a key mechanism in the study of the evolution of virulence. In the present situation, climatic variability impacts host availability and demography towards colonization by either of influenza virus or RSV. Given the size of the reproduction numbers that we have (that of RSV being larger than that of influenza), a very plausible explanation for the coexistence of both viruses in the system of reference is that RSV superinfects influenza allowing it to survive. The other case, influenza superinfecting RSV would result in the extinction of the inferior competitor ending up with a system with RSV epidemics only.

To support the model deduction that RSV could be thought of as an endemic, competitively dominant virus in SLP we can mention the following arguments: 1) for all age groups, mortality rate associated to RSV in the San Luis Potosí State is usually higher than influenza associated mortality rate [12], 2) human beings are the only reservoir of RSV implying that it must reside endemically in some geographical location to migrate to others (a sink-source dynamics as explained in [33, 39], in the case of the Influenza the diversity of strains generates highly dynamic transmission patterns where viral gene flow leads to the replacement of endemic viruses through competition for susceptible hosts [40–41], 3) it has been shown that in Mexico City and other cities at similar latitudes RSV is endemic, 4) RSV frequently reinfects the same individual during either the same or following seasons; humoral immunity against RSV seems to take about 3–5 years to become protective [12, 42–45].

**Fig 8:**
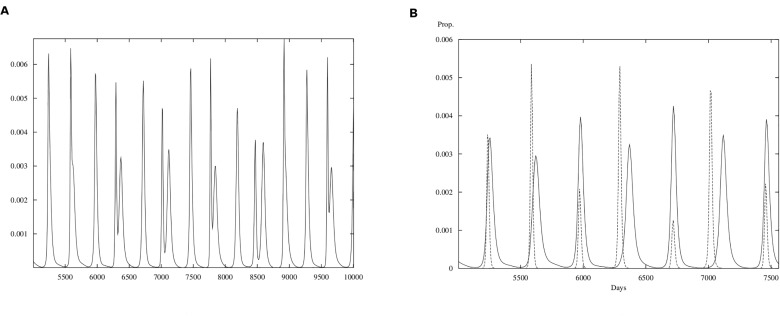
Simulations for the mathematical model. **(A)** Total cases (RSV plus influenza). **(B)** RSV cases are plotted with a continuous line; influenza cases plotted with a dashed line. Horizontal axis is time in days; vertical axis is proportion of the population. Plot is shown after a transient time of 5000 days where *σ* = 0.7, *b*_1_ = 0.9162 and *b*_2_ = 0.4566 and *θ* = 0.0001 for *R*_01_ = 1.83 and *R*_02_ = 2.28.

**Fig 9:**
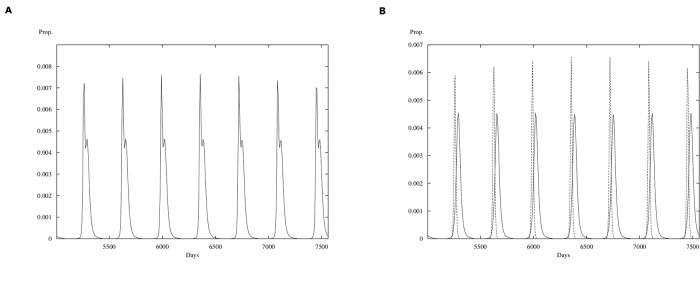
Simulations for the mathematical model. **(A)** Total cases (RSV plus influenza). **(B)** RSV cases are plotted with a continuous line; influenza cases plotted with a dashed line. Horizontal axis is time in days; vertical axis is proportion of the population. Plot is shown after a transient time of 5000 days where *σ* = 1, *b*_1_ = 0.9162 and *b*_2_ = 0.4566 and *θ* = 0.0001 for *R*_01_ = 1.83 and *R*_02_ = 2.28.

In Fig. 8 we show the biologically feasible case where RSV is competitively superior to influenza (*R*_01_ and *R*_02_ are the reproduction numbers for influenza and RSV respectively) and in Fig. 9 the same competitive dominance is illustrated but assuming that secondary infections occur as if the hosts had never been infected before, that is, *σ* = 1. The pattern in both figures is similar but that shown in Fig. 9 is much more regular. A decrease in *σ*, meaning a decrease in the contact rate for the secondary infection, provides a more irregular pattern both in the periodicity and the amplitude of the epidemic outbreaks in agreement with the recorded data. Tables 4 and 5 constitute the main support for RSV being the superior competitor. The magnitude of the estimated reproduction numbers have larger variability than those of influenza and the Tables show some evidence, however, week that years with *R*_0_ for RSV high are associated with *R*_0_ for influenza low. However, scarcity of data does not permit a conclusive statement. We will address this observation elsewhere.

**Fig 10:**
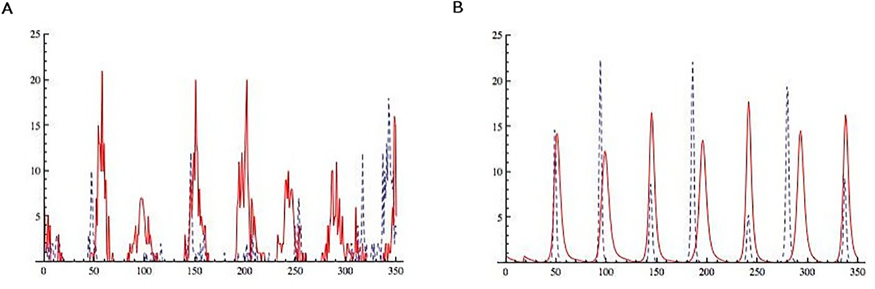
Real data and numerical simulations. RSV is plotted with a continuous line; influenza cases plotted with a dashed line. **(A)** RSV and influenza cases from SLP data. **(B)** RSV and influenza cases from model output. Horizontal axis is time in days; vertical axis is proportion of the population. Plot is shown after a transient time of 5000 days with arbitrary initial condition and parameters *σ* = 0.7, *b*_1_ = 0.9162 and *b*_2_ = 0.4566 and *θ* = 0.0001 for *R*_01_ = 1.83 and *R*_02_ = 2.28.

Before closing this section we present a last result that illustrates the qualities of the methodology used here to estimate parameters. In Fig. 10 we compare the dynamics of the observed outbreaks and the one coming out from numerical simulations of our model. Since in simulations we follow proportions and start our epidemics at arbitrary initial times and initial populations, we have shifted and rescaled the output from our model so the comparison has a better visual set up. We are not attempting to predict epidemic outbreaks but to explain the observed patterns. Note that not only the frequency of outbreaks is reproduced but also some qualitative features. For example, influenza epidemics have less amplitude and show interepidemic periods with zero or very low case numbers whereas RSV epidemics are broader and show and endemic phase between outbreaks. In S2 File (Figures B and C) we show different simulations for the cases *θ*_1_ ≠ *θ*_2_. In general we can observe that making the period of temporal immunity of the second virus shorter (*θ*_2_ larger), drives the model dynamics into a regime not supported by the data with periods, oscillations and amplitudes uncharacteristic compared to observed patterns. On the contrary the model predictions are relatively insensitive to changes in the duration of the immunity for the first virus.

## Discussion

**Fig 11:**
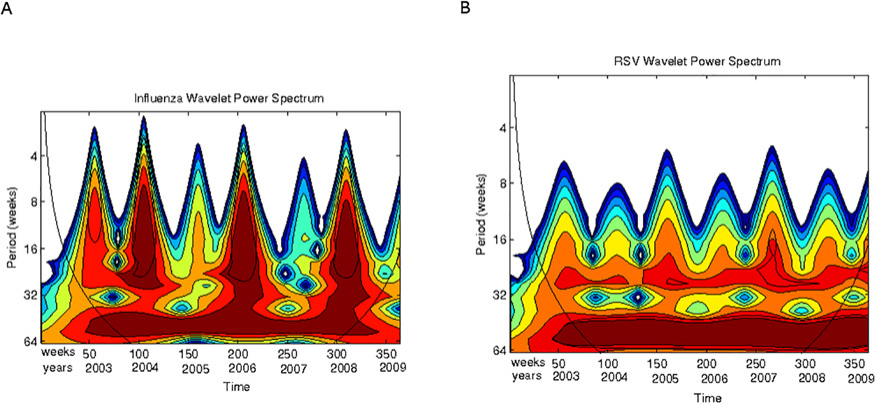
Scalogram (absolute values) for the model simulations for periods up to 64 weeks. **(A)** Influenza cases. **(B)** RSV cases. Horizontal axis, weeks numbered consecutively; vertical axis, periods in weeks. See text for explanation.

**Fig 12:**
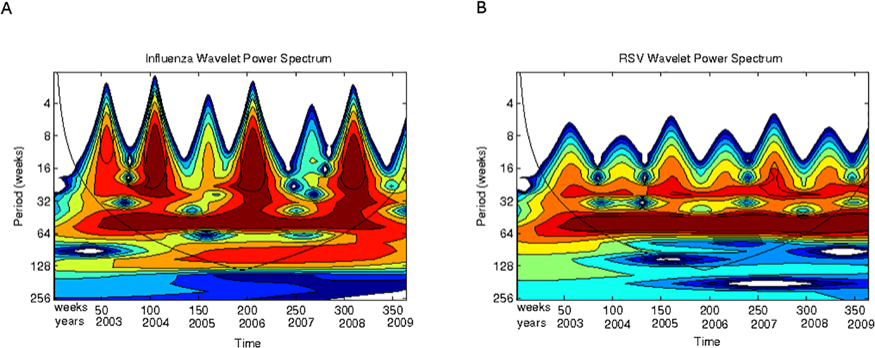
Scalogram (absolute values) for the model simulations for periods up to 128 weeks (32 months). **(A)** Influenza cases. **(B)** RSV cases. Horizontal axis, weeks numbered consecutively; vertical axis, periods in weeks. See text for explanation.

Now, to have a qualitative appreciation that the model encloses a plausible ecological explanation for the patterns of interactions observed in Mexico for RSV and influenza, the scalograms for model simulations are shown in Figs. 11 and 12. In Fig. 11 note that the power of the signal for influenza epidemics increases every two years whereas the power of RSV epidemics is weaker but essentially homogeneous each year. For both epidemics there is another cyclic behavior (besides the expected yearly one) at about 6 months (light band between 4 and 5 in the y-axis). Compare the scalogram for the real data presented in Fig. 5.

On the other hand, in Fig. 12 we show the scalogram but comprising a broader range of periods reaching up to 128 weeks. Note that influenza presents cyclic periodic behavior across all years between 64 and 128 weeks (on average every two years) whereas the signal for RSV is much more weaker for that same period. Compare with the scalograms for the real data shown in Fig. 6. In this case our model is a bit off since it generates periodicity slightly below that shown in the data (which is above 128 weeks). Nevertheless, one can see that, at least in terms of spectra, the similarities are remarkable.

Influenza comes in waves of discrete events since each new influenza epidemic is produced by a different viral strain. So there is a process of lineage or variant replacement which is a genetic process. This approach has been successfully investigated in [46]. Our approach looks at a different angle of the problem. We are interested in the interaction (or interference) between two types of virus: RSV and the influenza virus. In this case the process by which influenza occurs each year is due to an ecological process where available niches occupied by RSV are taken over and colonized by the influenza virus.

Our model is able to capture the two main cyclic behaviour observed in data: the annual one driven by yearly climatic variability which is exploited by RSV (since the power of its signal is stronger that of influenza) and a second biannual one, exploited by influenza. These two cyclic behaviours are in the root of the superinfection (ecological) mechanism for coexistence of both populations. It is important to mention that the data have a substantial bias in patient age that affects the estimates of reproduction number, that limits the extrapolation to the entire population. So a possible explication of the characteristics of scalograms is that the influenza is uncommon in children while RSV infects almost all the children during the first three years of life and re-infections by these viruses are very common during early childhood.

We must point out that our results presented here are out comes of, first, generating a better (still improvable) model for climatic variability. We renounced to the usual cosine contact rate with yearly period and instead took the temperature data and fitted a trigonometric series which was then incorporated in our model as described in the text above. Secondly, we estimated the reproductive numbers from the actual data. *R*_0_ estimation with such scarce data is a non-trivial but essentially methodological problem. There are many techniques that can be applied including likelihood and Bayesian approaches. However the point in all of them including the one we used here, rely on exploiting the data in such a way that one can get replicates that can improve our estimates.

Finally our choice for a model was a deterministic SEIRS one. The reason is very simple: we are dealing with seasonal influenza and RSV and there is certain immunity in a proportion of the population that reduces the number of susceptibles available for infection.

We must stress that we are not attempting to estimate the next outbreak of RSV or influenza nor attempting to statistically fit our model to the data. Our aim is strategic: we want a model that is able to explain the interaction observed between influenza and RSV through the analysis of broad patterns hidden in the time series. In order to have a better approximation to this program we certainly need to incorporate at least the role of asymptomatic infections, the role of immigration and a better way of introducing climatic variability into the equations.

## Supporting Information

S1 File Table A. Estimated values of *R*_0_ for first outbreak of influenza for a SIR model. Table B. Estimated values of *R*_0_ for second outbreak of influenza for a SIR model.
Table C. Estimated values of *R*_0_ for first outbreak of RSV.
Table D. Estimated values of *R*_0_ for second outbreak of RSV.
Table E. Estimation of *R*_0_ for ARIs data from SIR model using influenza parameters.
(PDF)

S2 File Figure A. Existence of equilibrium points as a function of the reproduction numbers. *P*_*j*_ denotes boundary equilibria where only one virus population exists; *P*_12_ denotes an interior equilibrium with both viral populations existing. The stability properties of each point were computed numerically using the values in Table 1 with influenza as virus 1 and RSV as virus 2.
**Figure B. Simulations for** $\hat{R}=R_{1}/R_{2}$ **for total cases (RSV plus influenza) and *θ*_2_ fixed.** The red line is when *θ*_1_ = *θ*_2_, green line stands *θ*_2_ > *θ*_1_ and blue line *θ*_1_ > *θ*_2_. For all cases *θ*_2_ = 0.0001.
**Figure C. Simulations for** $\hat{R}=R_{1}/R_{2}$ **for total cases (RSV plus influenza) and *θ*_1_ fixed.** The red line is when *θ*_1_ = *θ*_2_, green line stands *θ*_2_ > *θ*_1_ and blue line stands *θ*_1_ > *θ*_2_. For all cases *θ*_1_ = 0.00005.
(PDF)
