# Supplementary material for: Superinfection between Influenza and RSV Alternating Patterns in San Luis Potosí State, México
Source: PLoS One. 2015 Mar 24;10(3):e0115674. doi: 10.1371/journal.pone.0115674 (PMC4372574; doi:10.1371/journal.pone.0115674)
Supplement: S1 File — Table B. Estimated values of R 0 for second outbreak of influenza for a SIR model. Table C. Estimated values of R 0 for first outbreak of RSV. Table D. Estimated values of R 0 for second outbreak of RSV. Table E. Estimation of R 0 for ARIs data from SIR model using influenza parameters. (PDF) [file pone.0115674.s001.pdf]

Superinfection between influenza and RSV alternating patterns in San Luis Potosí State, México. SUPPLEMENTARY MATERIAL

J.X. Velasco-Hernández M. Núñez-López
A. Comas-García D. E. Noyola M. Capistrán

February 18, 2015

We calculated R_0 for both respiratory diseases using linear regression as described in the main text.

For Influenza and RSV we consider two outbreaks using the SIR model. In the following tables we list the estimated values for R_0 for both diseases and for ARIs.

Table A: **Estimated values of R_0 for first outbreak of influenza for a SIR model**

Year	R_0	β	*p-value*
2003	4.24	1.06	0.05
2004	—	—	—
2005	—	—	—
2006	3.67	0.92	0.74
2007	2.46	0.62	0.66
2008	2.21	0.55	0.05
2009	7	1.75	0.004

Table B: **Estimated values of R_0 for second outbreak of influenza for a SIR model**

Year	R_0	β	*p-value*
2003	2.67	0.67	0.03
2004	—	—	—
2005	2.97	0.74	0.008
2006	—	—	—
2007	—	—	—
2008	—	—	—
2009	6.09	1.52	0.049

Table C: **Estimated values of R_0 for first outbreak of RSV.**

Year	R_0	β	*p-value*
2003	—	—	—
2004	2.28	0.46	0.03
2005	—	—	—
2006	—	—	—
2007	—	—	—
2008	—	—	—
2009	4.75	2.38	0.33

Table D: **Estimated values of R_0 for second outbreak of RSV**

Year	R_0	β	*p-value*
2003	2.26	0.47	0.04
2004	8.9	1.78	0.005
2005	4.07	0.82	0.002
2006	2.45	0.49	0.02
2007	5.78	1.16	0.0003
2008	6.32	1.26	0.0008
2009	—	—	—

Table E: **Estimation of R_0 for ARIs data from SIR model using influenza parameters**

Year	R_0	β	*p-value*
2000	1.0035	0.2508	0.001
2001	1.0030	0.2507	0.003
2002	1.0034	0.2508	0.017
2003	1.0040	0.2510	0.005
2004	1.0067	0.2516	0.0005
2005	1.0078	0.2519	4.4e-5
2006	1.0064	0.2516	7.0e-10
2007	1.0023	0.2505	0.003
2008	1.0039	0.2509	2.0e-6
2009	1.0012	0.2503	0.0009
2010	1.0040	0.2510	1.4e-5

In figure 1 of the manuscript we show ARIs data from 2000-2010, so we present the replacement number R_e defined as the expected number of secondary infections that one infected person would produce through the entire duration of the infectious period (where the population need not be fully susceptible) for the total ARIs data; this is because all population is not susceptible at the beginning of each year [44].

Assuming that virus 1 is influenza and virus 2 is RSV, the replacement number of model (1) for each virus is given by

$$R_{e1} = \frac{\beta_1\gamma_1}{(\mu+\eta_1)(\mu+\gamma_1)}(S+R_2), \qquad R_{e2} = \frac{\beta_2\gamma_2}{(\mu+\eta_2)(\mu+\gamma_2)}(S+E_1+I_1+R_1)$$

where R_{e1} and R_{e2} are the replacement numbers for influenza and RSV respectively and with parameters $\sigma = 0.7$, $b_1 = 0.9162$ and $b_2 = 0.4566$ we obtain $R_{e1} = 3.62$ and $R_{e2} = 0.07$. Given the size of the basic reproduction and replacement numbers for RSV case so if $R_{e2} < 1$ for $R_{02} > 1$ either through an increase in the infectious population or the recovered population; in this case increases the infectious population of RSV and according to the model this makes it possible to infer the coexistence of both viruses where RSV superinfects influenza allowing it to survive.
