# Supplementary material for: Superinfection between Influenza and RSV Alternating Patterns in San Luis Potosí State, México
Source: PLoS One. 2015 Mar 24;10(3):e0115674. doi: 10.1371/journal.pone.0115674 (PMC4372574; doi:10.1371/journal.pone.0115674)
Supplement: S2 File — P j denotes boundary equilibria where only one virus population exists; P 12 denotes an interior equilibrium with both viral populations existing. The stability properties of each point were computed numerically using the values in Table 1 with influenza as virus 1 and RSV as virus 2. Figure B. Simulations for R^=R1/R2 for total cases (RSV plus influenza) and θ2 fixed. The red line is when θ 1 = θ 2, green line stands θ 2 > θ 1 and blue line θ 1 > θ 2. For all cases θ 2 = 0.0001. Figure C. Simulations for R^=R1/R2 for total cases (RSV plus influenza) and θ1 fixed. The red line is when θ 1 = θ 2, green line stands θ 2 > θ 1 and blue line stands θ 1 > θ 2. For all cases θ 1 = 0.00005. (PDF) [file pone.0115674.s002.pdf]

Superinfection between influenza and RSV alternating patterns in San Luis Potosí State, México. SUPPLEMENTARY MATERIAL

J.X. Velasco-Hernández   M. Núñez-López
A. Comas-García   D. E. Noyola   M. Capistrán

February 18, 2015

The SEIRS model for diseases has a long history and has been amply analyzed. Here we will limit ourselves to the determination of equilibrium points and a local stability analysis and numerical simulations of relevant characteristics. In this section we consider only the case where the contact rates β_j are constant. To ease the mathematical analysis we consider the case where $\theta_1 = \theta_2$ and then illustrate the consequences of the inequality numerically.

The phase space of our model is the subset of the non-negative cone

$$\Omega = \{(S, E_1, I_1, R_1, E_2, I_2, R_2) \in R^7 : 0 \leq S+E_1+I_1+R_1+E_2+I_2+R_2 \leq 1\}.$$

Applying the next-generation matrix methodology (Velasco-Hernández, 1994) we obtain that the reproduction number for the system is

$$R_0 = \max\{R_{01}, R_{02}\},$$

where

$$R_{0j} = \frac{\beta_j \gamma_j}{(\mu + \eta_j)(\mu + \gamma_j)} \qquad j = 1, 2.$$

The disease-free equilibrium exists and it is given as $P_0 = (1, 0, 0, 0, 0, 0, 0)$.

The boundary equilibria (those equilibrium points where only one of the diseases exist) can be computed from the equations. Here we only write the

expressions for the infectious states I_1^* and I_2^* since they play a role in the discussion that follows:

$$I_1^* = \frac{(\mu+\theta)^2(\mu+\eta_1)(R_{01}-1)}{\beta_1(\mu(\theta+\mu)(\mu+\eta_1)+\gamma_1(\theta+\mu+\eta_1))},$$

$$I_2^* = \frac{\mu(\mu+\theta)^2(\mu+\eta_2)(R_{02}-1)}{\beta_2((\mu+\gamma_2)(\theta+\mu)(\mu+\eta_2)-\gamma_2\theta\eta_1)}.$$

Note that the denominator of I_2^* depends on η_1. We will denote the equilibrium point where only virus j is present as P_j.

To determine the existence of an interior equilibrium point (where both viruses are present in the system) we equate the right-hand side of our equations to zero and, after some algebraic manipulations, we can obtain expressions for the coordinates of the equilibrium points. The existence, uniqueness and positivity of any of the possible interior equilibria present difficulties due to the number of parameters that the model has. However, we can explore an special case and from it we will attempt a generalization.

Set $\sigma = 1$. This is interpreted as assuming that the secondary infection takes place with contact rates identical to those recorded for infections of naive hosts. After some algebraic manipulation we can see that I_1^{**} depends on the reproduction numbers of both viruses whereas I_2^{**} depends only of a single parameter of the other virus: η_1. In other words, changes in the parameters related to virus 1 have little, if any, effect on the density of I_2 at equilibrium whereas changes in the parameters for virus 2 can have a large effect on the equilibrium value and even on the biological feasibility of I_1.

To find the interior equilibrium point we reduce the original seven equations to a system of two bivariate functions

$$m_1(I_1, I_2) = 0, \qquad m_2(I_1, I_2) = 0$$

whose solutions give the coordinates (I_1^{**}, I_2^{**}) of the interior equilibrium point. Following backwards the process of simplification done to obtain the rational functions m_1 and m_2 we can recover the rest of the coordinates. If I_1^{**} and I_2^{**} are positive, so are the rest of the coordinates. For the special case $\sigma = 1$ one can solve the system explicitly to obtain that

$$I_1^{**} = I_1^{**}(R_{01}, R_{02}), \qquad I_2^{**} = I_2^{**}(\eta_1).$$

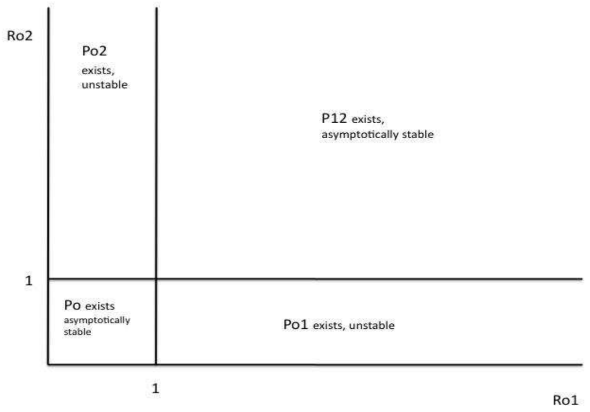

Figure A: **Existence of equilibrium points as a function of the reproduction numbers.** P_j denotes boundary equilibria where only one virus population exists; P_{12} denotes an interior equilibrium with both viral populations existing. The stability properties of each point were computed numerically using the values in Table 1 with influenza as virus 1 and RSV as virus 2.

Using the expression for the boundary equilibrium for I_2^*, we can see that its existence depends on two conditions: the first, the typical one, is that $R_{02} > 1$, the second however is interesting, see Figure A.

Now we will explore numerically the consequences of assuming the more realistic scenario of having two different durations of immunity. Using the baseline value of $\theta = 0.0001$ (see main text) we verified the effect that a different value of θ_1 has on the overall dynamics of the epidemics. In Figure B a lower value of θ_1 conserves the frequency of the oscillations (but not the amplitude which is lower than before). Taking higher values of θ_1 essentially preserved the frequency and makes the amplitude more regular. In Figure C we show the dynamics with a much smaller base line immunity value for the superior competitor. Here the dynamics is very much different to the observed in the previous case and in the data. Thus, the qualitative dynamical behavior of the model does not change under the first scenario described above which is the one of interest in this work.

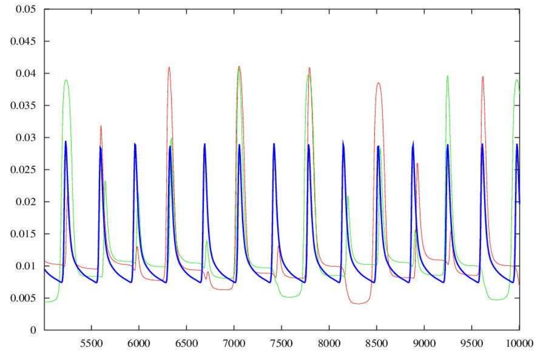

Figure B: **Simulations for $\hat{R} = R_1/R_2$ for total cases (RSV plus influenza) and θ_2 fixed.** The red line is when $\theta_1 = \theta_2$, green line stands $\theta_2 > \theta_1$ and blue line $\theta_1 > \theta_2$. For all cases $\theta_2 = 0.0001$.

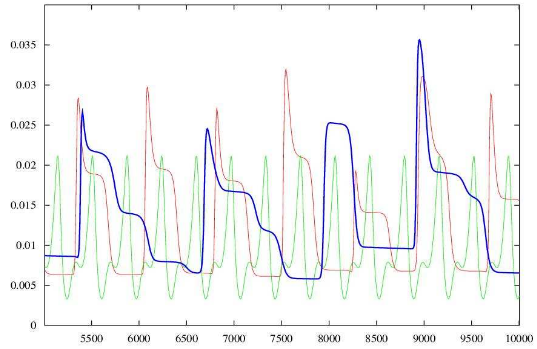

Figure C: **Simulations for $\hat{R} = R_1/R_2$ for total cases (RSV plus influenza) and θ_1 fixed.** The red line is when $\theta_1 = \theta_2$, green line stands $\theta_2 > \theta_1$ and blue line stands $\theta_1 > \theta_2$. For all cases $\theta_1 = 0.00005$.

Finally, to obtain the forcing function $f(t)$ that multiplies the contact rate β_k we obtained the data for daily temperatures and adjusted a trigonometric polynomial to it resulting in the function

$$\begin{aligned} f(t) &= 17.33 + 0.480 \sin\left[\frac{2\pi}{365}t\right] - 0.40 \sin\left[\frac{2\pi}{365}2t\right] - 4.03 \cos\left[\frac{2\pi}{365}t\right] \\ &\quad -1.05 \cos\left[\frac{2\pi}{365}2t\right] \end{aligned}$$

In supplement 1 we are estimating the reproduction number for each outbreak assuming that the time scales of infection and recovery are significantly shorter than the time scale of the environmental fluctuation. This assumption allows us to zoom in on each outbreak and treat it as an isolated event where the demographic and epidemiological processes dominate over the environmentally driven oscillations.
